# Standard Anatomic Terminologies: Comparison for Use in a Health Information Exchange–Based Prior Computed Tomography (CT) Alerting System

**DOI:** 10.2196/medinform.8765

**Published:** 2017-12-14

**Authors:** Anton Oscar Beitia, Tina Lowry, Daniel J Vreeman, George T Loo, Bradley N Delman, Frederick L Thum, Benjamin H Slovis, Jason S Shapiro

**Affiliations:** ^1^ Department of Emergency Medicine Icahn School of Medicine at Mount Sinai New York, NY United States; ^2^ Center for Biomedical Informatics Regenstrief Institute Indianapolis, IN United States; ^3^ Department of Medicine Indiana University School of Medicine Indianapolis, IN United States; ^4^ Department of Radiology Icahn School of Medicine at Mount Sinai New York, NY United States; ^5^ Department of Emergency Medicine Thomas Jefferson University Philadelphia, PA United States

**Keywords:** tomography, x-ray computed, health information exchange, radiation dosage, terminology, anatomy, regional

## Abstract

**Background:**

A health information exchange (HIE)–based prior computed tomography (CT) alerting system may reduce avoidable CT imaging by notifying ordering clinicians of prior relevant studies when a study is ordered. For maximal effectiveness, a system would alert not only for prior same CTs (exams mapped to the same code from an exam name terminology) but also for similar CTs (exams mapped to different exam name terminology codes but in the same anatomic region) and anatomically proximate CTs (exams in adjacent anatomic regions). Notification of previous same studies across an HIE requires mapping of local site CT codes to a standard terminology for exam names (such as Logical Observation Identifiers Names and Codes [LOINC]) to show that two studies with different local codes and descriptions are equivalent. Notifying of prior similar or proximate CTs requires an additional mapping of exam codes to anatomic regions, ideally coded by an anatomic terminology. Several anatomic terminologies exist, but no prior studies have evaluated how well they would support an alerting use case.

**Objective:**

The aim of this study was to evaluate the fitness of five existing standard anatomic terminologies to support similar or proximate alerts of an HIE-based prior CT alerting system.

**Methods:**

We compared five standard anatomic terminologies (Foundational Model of Anatomy, Systematized Nomenclature of Medicine Clinical Terms, RadLex, LOINC, and LOINC/Radiological Society of North America [RSNA] Radiology Playbook) to an anatomic framework created specifically for our use case (Simple ANatomic Ontology for Proximity or Similarity [SANOPS]), to determine whether the existing terminologies could support our use case without modification. On the basis of an assessment of optimal terminology features for our purpose, we developed an ordinal anatomic terminology utility classification. We mapped samples of 100 random and the 100 most frequent LOINC CT codes to anatomic regions in each terminology, assigned utility classes for each mapping, and statistically compared each terminology’s utility class rankings. We also constructed seven hypothetical alerting scenarios to illustrate the terminologies’ differences.

**Results:**

Both RadLex and the LOINC/RSNA Radiology Playbook anatomic terminologies ranked significantly better (*P*<.001) than the other standard terminologies for the 100 most frequent CTs, but no terminology ranked significantly better than any other for 100 random CTs. Hypothetical scenarios illustrated instances where no standard terminology would support appropriate proximate or similar alerts, without modification.

**Conclusions:**

LOINC/RSNA Radiology Playbook and RadLex’s anatomic terminologies appear well suited to support proximate or similar alerts for commonly ordered CTs, but for less commonly ordered tests, modification of the existing terminologies with concepts and relations from SANOPS would likely be required. Our findings suggest SANOPS may serve as a framework for enhancing anatomic terminologies in support of other similar use cases.

## Introduction

### Background

Computed tomography (CT) use has grown dramatically in recent years [1,2] and, because CT typically delivers higher radiation doses than conventional x-rays, there are concerns about appropriateness of utilization and the risks of cumulative radiation exposure [1,3-5]. Prior work by our group showed many patients underwent the same CT exam at more than one site within a health information exchange (HIE); some were likely duplicate studies and possibly avoidable [6]. Other authors have also shown that many CTs are duplicative and may be unnecessary [1,7-12].

An HIE-based prior CT alerting system may reduce avoidable CT imaging by notifying ordering clinicians of prior relevant studies when a repeat study is ordered [9,10,13,14]. For maximal effectiveness, a system would alert not only for prior *same* CTs (exams mapped to the exact same code from an exam name terminology) but also for *similar* CTs (exams in the same anatomic region but with different CT protocols and mapped to different exam name terminology codes) and anatomically *proximate* CTs (exams in adjacent anatomic regions). For example, in such an alerting system, a clinician ordering a head CT without intravenous contrast would be alerted not only to the same prior exam but also to a prior *similar* head CT with contrast study or a prior *proximate* neck CT performed anywhere within an HIE. Alerts for prior *proximate* CTs may be beneficial as scans often extend to tissues beyond the nominal scan range [15-17].

Alerting based on the existence of other prior imaging modalities (magnetic resonance imaging [MRI], ultrasound, plain films, and nuclear medicine) may also have utility in the decision to order a CT or other new imaging study. Although inclusion of all imaging modalities is the ultimate goal in our alerting system, we decided to start first with CT studies because of their frequency of use, cost, and potential impact on patient safety because of the relatively higher amount of radiation.

Notification of previous *same* studies across an HIE requires mapping of local site CT codes to a standard terminology for exam names (such as Logical Observation Identifiers Names and Codes [LOINC]) to show that two studies with different local codes and descriptions are equivalent (eg, “CT (-) head” at one site and “CT brain w/o contrast” at another) [18]. Notifying of prior *similar* or *proximate* CTs requires an additional mapping of LOINC CT codes to anatomic regions, ideally coded by an anatomic terminology. Several anatomic terminologies exist, including the anatomic hierarchy contained in LOINC’s multiaxial hierarchy, but no prior studies have evaluated how well the concepts and relationships in these terminologies would support the alerting use case.

### Objective

We sought to evaluate the fitness of five existing standard anatomic terminologies to support our alerting use case, including the (1) Foundational Model of Anatomy (FMA), (2) Systematized Nomenclature of Medicine Clinical Terms (SNOMED CT), (3) Radiological Society of North America’s (RSNA) RadLex anatomic terminology, (4) anatomic hierarchy associated with LOINC, and (5) LOINC/RSNA Radiology Playbook’s anatomic terminology by comparing them with an anatomic framework that we created specifically to meet the operational needs of our use case: “Simple ANatomic Ontology for Proximity or Similarity” (SANOPS).

We did not create SANOPS as a new anatomic framework for general use. Rather, we aimed to create a simple anatomic framework that could be implemented easily to support our alerting application and could also be used as a reference by which to compare the fitness for use of other existing terminologies. Our goals were to determine whether any of the existing terminologies could perform adequately in an unaltered state for our specific application and to characterize where they could be enriched, if necessary.

### Significance

This study is a novel investigation of anatomic terminologies to support a prior CT alerting system. We previously described the pilot work to conceive of the SANOPS anatomic framework, which arose because we were designing an alerting system that accounted for similar and proximate CTs [19]. Other authors have compared the anatomic representations of SNOMED CT and FMA [20] and used anatomic terminologies to support radiology applications [21-23]. There are also reports of CT alerting systems implemented to notify ordering clinicians of an exam’s appropriateness [24,25], as well as to notify technologists of possible excessive patient radiation dose [26]. These prior studies were not performed in the context of a prior CT alerting system and did not use an HIE of multiple organizations as the data source.

## Methods

### Overview

As an overview, to compare the existing standard terminologies, we previously developed an idealized anatomic framework (SANOPS) that we would use as a reference. In this study, we devised a terminology utility classification to provide a quantitative assessment for the effort required to utilize and implement the standard anatomic terminologies for our use case compared with the SANOPS benchmark. We mapped a sampling of LOINC CT exam codes extracted from our regional HIE to anatomic regions in each terminology and assigned utility classes to each terminology for each mapping (described in detail below). We then statistically compared the utility classes of each terminology. We also constructed seven hypothetical alerting scenarios to further illustrate the terminologies’ differences.

We performed our terminology comparison from December 2015 to February 2016. The versions of the terminologies were the latest available at the time and are indicated below.

### Materials: Optimal Anatomic Terminology Features and SANOPS

Our development of SANOPS was guided by anatomic requirements and unique operational challenges required to support our specific use case of issuing prior *similar* or *proximate* alerts in an HIE-wide prior CT alerting system.

Anatomically, to support *similar* or *proximate* alerts, our ideal anatomic terminology would be organized by body regions rather than by organ systems and have information regarding containment of organs. For example, to issue a *similar* alert of a prior liver CT with a kidney CT order requires only that the terminology has information that the kidney and liver are contained in the same anatomic region, that is, the abdomen. To issue a *proximate* alert of a prior kidney CT with a pelvis CT order requires information that the kidney is contained in the abdomen region and that the abdomen and pelvis are specified as adjacent body regions. A terminology where kidneys are nested under the genitourinary system, without links to abdomen, would not be suitable. If a terminology is organized in an organ system hierarchy, then to be of any use it should also have information regarding the containment of organs within body regions. For the extremities, we would prefer a division into at least three relatively equivalent-sized proximate, mid, and distal anatomic regions. This would be done to help avoid clinically irrelevant proximate or similar alerts. For example, a prior right foot CT should not trigger an alert when a new right hip CT is ordered.

Operationally, issuing an order-time alert based on a prior exam performed at a different site within an HIE requires a complex series of steps. The local site where the order is placed must issue a Web-based communication with the HIE server that must match patient and exam records and determine whether same, similar, or proximate exams exist and then return the alert result and payload back to the local site. To be of any practical value, these steps must all be completed within fractions of a second. Any step that conserves computational resources and reduces query time would help ensure the successful firing of such an alert within the clinician’s workflow. Given these special circumstances, it follows that body region organization and organ containment information within body regions should be expressed in the most direct and simple fashion as possible.

**Figure 1:**
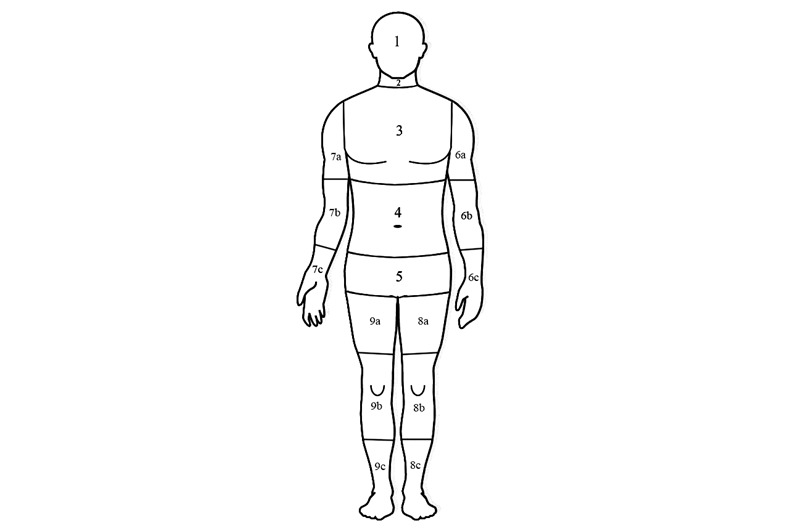
Seventeen major anatomic regions defined by our novel Simple ANatomic Ontology for Proximity or Similarity (SANOPS) terminology. The major anatomic regions and their respective codes are: 1-head, 2-neck, 3-chest, 4-abdomen, 5-pelvis, 6a-proximal left upper extremity (LUE), 6b-mid LUE, 6c-distal LUE, 7a-proximal right upper extremity (RUE), 7b-mid RUE, 7c-distal RUE, 8a-proximal left lower extremity (LLE), 8b-mid LLE, 8c-distal LLE, 9a-proximal right lower extremity (RLE), 9b-mid RLE, and 9c-distal RLE.

With these anatomic requirements and operational issues in mind, we designed the SANOPS anatomic framework with a relatively simplistic design. SANOPS divides the human body into 17 major regions (Figure 1). We used SANOPS by linking CT exam codes (which are LOINC codes in our application) to body regions that best subsume the region imaged. For example, kidney CT would be assigned to the abdominal region. Multiple anatomic identifiers can be assigned to exams that span more than one major body region. For example, an abdomen and pelvis CT exam as well as a lumbar spine CT exam would both be assigned to the abdomen and pelvic regions. Using SANOPS regions, similar alerts would be issued for a pair of different LOINC CT codes when they mapped to the same body regions. Proximate alerts would be issued for two CT codes that are assigned adjoining SANOPS regions, providing that they are not also assigned to the same region (in which case a similar alert would be issued).

To avoid clinically irrelevant proximate or similar alerts in the extremities, SANOPS divides extremities into proximal, mid, and distal portions, with midshafts of long bones separating these regions. Therefore, extremity regions roughly correspond to the respective large joints plus adjacent portions of long bone shafts. This approach differs from the other anatomic terminologies that divide upper extremities into arm, forearm, and hand and wrist regions and the lower extremities into thigh, leg, and foot and ankle regions.

Our long-term goal is that SANOPS informs the use of standard terminologies to support HIE-wide prior exam alerting systems. More information regarding SANOPS and a translation table of SANOPS codes to other existing terminologies is available on the Internet [27].

### Materials: Anatomic Terminologies

The FMA is an open source reference domain anatomic terminology of over 75,000 distinct anatomic concepts covering material objects from macroscopic to microscopic level, as well as nonmaterial entities (such as anatomic spaces) [28]. The FMA is both broader and more granular than extant anatomy texts or other terminologies. The FMA is an ontology in that it is “concerned with the representation of classes or types and relationships necessary for the symbolic representation of the phenotypic structure of the human body in a form that is understandable to humans and is also navigable, parseable, and interpretable by machine-based systems [29].” The FMA organizes its anatomic taxonomy in strict subsumption hierarchy. The FMA’s anatomic structural abstraction also contains partonomy information that relates organ systems to constituent parts through *part_of*, *constitutional_part_of*, and *regional_part_of* links [29]. Many instances of FMA’s *part_of* links relate organ systems to anatomic body regions. We used FMA version 4.4.1.

RadLex is a publicly available comprehensive clinical terminology providing a uniform standard for all radiology-related information [30]. The version used (3.13) contained over 68,000 terms organized in 15 main categories, including anatomic entity, clinical finding, and imaging modality. RadLex’s anatomic terminology is derived from the FMA but employs simplified macroscopic terms relevant to radiology [31]. The RadLex Playbook (version 2.0 studied), comprising part of the comprehensive RadLex terminology, is a catalogue of radiology orderable exams, each given a unique “*RadLex* Playbook identifier (RPID)” defined by several attributes, including modality, body region, and anatomic focus. Body part(s) are indicated through body region and anatomic focus attributes that are represented by concepts in RadLex’s anatomic terminology. Body region specifies the broad portion of the body that is imaged and anatomic focus indicates a more specific location (ie, liver CT has body region attribute “abdomen” and anatomic focus “liver”) [32].

LOINC is a freely available international standard developed by the Regenstrief Institute Inc for tests, measurements, and documents. The version used (2.54) contained 78,959 terms with 798 CT exam codes [33]. Each radiology code has a system attribute (part) corresponding with the region or organ on which that exam was performed. LOINC anatomic regions are arranged hierarchically, although formal rules and relations between classes and subclasses are not currently defined.

The LOINC/RSNA Radiology Playbook was initially released in December 2015 as part of LOINC version 2.54 and was developed through collaboration between Regenstrief Institute and the RSNA and with support from the National Institute of Biomedical Imaging and Bioengineering [34]. This no-cost product combines and unifies useful aspects of LOINC Radiology and the RadLex Playbook. We used the initial release version that was limited to CT. Subsequent releases (most recent June 2017; part of LOINC version 2.61) included MRI, x-ray, ultrasound, nuclear medicine, mammography, and other imaging modalities [35]. Similar to RadLex, each CT exam in the LOINC/RSNA Radiology Playbook is defined by several attributes. Body part attributes are drawn from the RadLex anatomic hierarchy, with region imaged and imaging focus attributes in many instances also following RadLex’s body region and anatomic focus model [35]. Where applicable, matching RadLex RPID codes are mapped to the LOINC codes.

SNOMED CT is a clinical health care terminology originally created by the College of American Pathologists and now maintained by SNOMED International (formerly, the International Health Terminology Standards Development Organisation) [36]. The version used (September 2015 release) is comprised of about 300,000 concepts, of which over 30,000 pertain to anatomic structures. SNOMED’s anatomic hierarchy uses a Structure-Part-Entire (SEP) triplet to represent anatomic entities (eg, liver is represented through concepts of liver part, entire liver, and liver structure) [37]. This allows for anatomic relations to be expressed as subsumption (*is_a*) relations rather than *part_of* relationships (eg, coronary artery structure *is_a* heart part *is_a* heart structure). SNOMED CT also contains direct partonomy information with anatomic structures related to constituent parts through *part_of* links that parallel the SEP relations [38]. By design, SNOMED CT’s anatomic hierarchy is also a polyhierarchy, with many anatomic concepts having multiple parents or ancestors and children or descendants [38].

### Methods: Mapping LOINC CT Exam Codes to Anatomic Regions in Anatomic Terminologies

#### Overview

The Office of the National Coordinator for Health IT has recommended LOINC as the best available standard for imaging procedures [39], and it has been used successfully in large HIEs [40]. Healthix, a large New York City area HIE currently working with our group, has chosen LOINC as the exam name terminology standard for CT exams and is mapping all local institution exam codes to LOINC. For these reasons, we chose LOINC as the exam name terminology to provide standardized CT exam codes and descriptions to which we assigned or mapped anatomic regions from our candidate anatomic terminologies, including LOINC’s own core anatomic hierarchy. We extracted 100 random LOINC CT exam codes and the 100 most frequently performed LOINC CT exam codes among five sites in Healthix from March 1, 2009 to July 24, 2012. One of the authors (AOB), a board-certified radiologist, informatician, and domain expert manually mapped the anatomic regions from the LOINC name to the candidate anatomic terminologies. Our sampling approach enabled assessment of anatomic terminology performance over both a random sample and CTs that users would most frequently encounter.

#### Mapping Approach

LOINC CT exam codes were mapped to each anatomic terminology’s region representing the nearest match to the region specified in the LOINC long common name. For example, “Head CT” and “Kidney CT” were respectively mapped to the “head” and “kidney” classes in FMA and were mapped to “head structure” and “kidney structure” concepts in SNOMED CT.

We made special considerations in mapping the LOINC CT exam codes to the LOINC, RadLex, and LOINC/RSNA Playbook anatomic terminologies because they are parts of larger comprehensive terminologies that include standardized codes and names or descriptions for CT exams and already have anatomic region attributes embedded with their CT exam names.

We mapped LOINC CT exam codes to regions in LOINC’s core anatomic terminology by linking to the anatomic *system* attribute specified in the LOINC fully specified name [34]. Similarly, we linked LOINC CT exam codes to anatomic regions in the LOINC/RSNA Playbook anatomic terminology through the *region imaged* and *imaging focus* attributes contained in that terminology's distribution file. We also leveraged attribute relationships in the LOINC/RSNA Playbook to link LOINC CT exam codes to RadLex’s core anatomic terminology through mappings between LOINC CT codes and RadLex Playbook RPID codes. If a LOINC code had no matching RPID, one of the authors (AOB) chose the closest matching RPID through manual review of the RadLex Playbook. Once we identified the corresponding RadLex Playbook RPID, we mapped LOINC Codes to each RPID’s *body region* and *anatomic focus* attributes.

To further illustrate our method of anatomic mapping, consider the “Liver CT” exam (LOINC code 24815-3). In LOINC’s anatomic terminology, we use “Abdomen>Liver” as the anatomic region because this composite element is the *system* attribute for this LOINC exam code. In both the LOINC/RSNA Radiology Playbook and RadLex anatomic terminologies, the anatomic mappings were to *body region* of “abdomen” and *anatomic focus* of “liver,” as these were specified as attributes of the exam code in both terminologies. For FMA, the exam was mapped to *anatomic class* of “liver.” For SNOMED CT, the exam code was mapped to *anatomic concept* of “liver structure.”

#### Evaluation With Anatomic Terminology Utility Classification

##### Anatomic Terminology Utility Classification Features

Once a LOINC CT exam code was mapped to anatomic regions in each standard anatomic terminology, a terminology utility class was assigned to each mapping. We devised an anatomic terminology utility classification to provide a structured assessment of the effort required to utilize and implement the standard anatomic terminologies in an unmodified state for our use case. It is an ordinal sliding scale from 1 to 5. The criteria used to assign a terminology utility class is summarized in Table 1 and discussed in detail below. Using SANOPS as a benchmark, the class assignment is based on an approximation of terminology modifications or additional computing steps required to use the standard terminology to support an appropriate *similar* or *proximate* alert; a class of “1” is given if no additional computing steps above those used with SANOPS are necessary for a terminology to fully support an appropriate alert in its unaltered state; and a class of “5” is assigned if a terminology requires a large amount of computing resources or modifications. Utility class assignment was performed manually by AOB and then reviewed and validated by the other coauthors.

**Table 1:** Summary of terminology utility classification scale for mapping of each anatomic terminology to Logical Observation Identifiers Names and Codes (LOINC) coding.

Class	Criteria
1	The anatomic region specified is a body region (not an anatomic focus) that maps to a region in SANOPS^a^ *or* if an anatomic focus, the corresponding body region attribute from the candidate terminology, maps to a SANOPS region
2	Anatomic focus nested under a SANOPS body region in uniform relation without other alternative path(s)
3	Polyhierarchy with uniform type (“is-a” or “part-of”) edge links from anatomic focus concept to SANOPS body region but also with other paths to other superclasses bypassing the SANOPS body region
4	No link from anatomic focus to SANOPS body region through single type of edge relation
5	Anatomic focus concept not nested under SANOPS body region

^a^SANOPS: Simple ANatomic Ontology for Proximity or Similarity.

###### Anatomic Terminology Utility Classification: Class of “1”

A class of “1” was given when the anatomic region in the target terminology required no further computing steps above those if SANOPS were used to support a prior *similar* or *proximate* exam alert. This was satisfied in the following conditions:

- When the anatomic region specified by the LOINC CT exam name was equivalent to a SANOPS-specified major body region. For example, mappings of “Abdomen and Pelvis CT” in all five candidate terminologies were assigned “1” because all have concepts for abdomen and pelvis and there is a one-to-one correspondence of regions specified in the exam name with anatomic concepts in each terminology.
- For LOINC, RadLex, and LOINC/RSNA Playbook, when the *body region* or *imaged region* attribute specified by the exam code corresponded with one of SANOPS major body regions. For example, for a “Neck vessel CT angio” a class of “1” was given to RadLex and the LOINC/RSNA Radiology Playbook as “neck” is specified as a body region attribute in exam codes of both terminologies.
- In the extremities, when the anatomic region specified by the LOINC CT exam name was a major joint and the terminology had a matching concept for the specified joint. For example, mappings of “Right Knee CT” in all five candidate terminologies received a class rank of “1” because all have “right knee” anatomic concepts. The rationale for this assignment is that SANOPS concepts of proximal, mid, and distal regions of the extremities roughly corresponded with large extremity joints plus adjacent long bone shafts.
- If the CT exam description specified an entire extremity only as the anatomic region imaged and the terminology had a corresponding anatomic concept for the entire extremity.

###### Anatomic Terminology Utility Classification: Class of “2”

A terminology utility class of “2” was given to a target terminology if the anatomic focus specified by the CT exam code was nested directly under a major body region in SANOPS via uniform relationships and without other alternative path(s). For example, for a “CT angio abdomen,” LOINC’s anatomic terminology was assigned a class of “2” as the “abdominal vessels” anatomic region was nested directly under “abdomen.” The rationale for this assignment is that this relation can be expressed in a “look-up” table and can be queried simply and quickly with only slightly more computing time and resources required over using SANOPS alone.

###### Anatomic Terminology Utility Classification: Class of “3”

A class of “3” was given to a target terminology in cases of a polyhierarchy where there were uniform type edge links from anatomic focus concept specified in the CT code to a body region corresponding with one of the SANOPS body regions, but also, there were paths to other superclasses bypassing the SANOPS body regions. For example, SNOMED CT ranked a “3” for “Temporal bone CT,” as there were direct “ *is_a”* links to the “head” through some paths, but there were also other “ *is_a”* paths linking to the skeletal system, bypassing “head.” The rationale for this assignment is that although this class-subclass or parent-child relation can also be expressed in a simple “look-up” table, the presence of multiple parents can lead to errors in classification, and description logic reasoners may need to be applied to ensure and verify that all anatomic class relations are expressed prior to implementation.

###### Anatomic Terminology Utility Classification: Class of “4”

A class of “4” was given to a target terminology when multiple different types of edge relations were necessary to link back to major anatomic regions. For example, FMA ranked a “4” for “Esophagus CT” because reaching the body region of chest requires first traveling down *has_part* link to thoracic esophagus and then back through *part_of* links to arrive at chest. The rationale for this class assignment is that to link back to major anatomic region through multiple different edge links would likely require a relatively complex algorithm and would require considerably more computer resources and processing time over using SANOPS alone.

###### Anatomic Terminology Utility Classification: Class of “5”

A class of “5” was given if there are no relationships to a major body region in the terminology from the anatomic focus specified by the exam code. For example, a class of “5” was assigned to LOINC and RadLex’s terminologies for “Cervical spine CT” because each lacked links from “cervical spine” to “neck” and in neither was neck specified as a body region attribute by the exam code. The rationale for this class assignment is that the terminology could not be used in its unmodified state to support our use case. Special modifications would be needed to link the anatomic focus to the major SANOPS body region.

##### Analysis of Anatomic Terminology Utility Classes

Descriptive statistics were performed on the anatomic terminology utility classes for each terminology. As the data were ordinal, median and mode terminology utility classes for each of the five candidate terminologies were calculated. Mean classes and standard deviations for each of the five candidate terminologies were also calculated, although these are less informative for ordinal data. The Kruskal-Wallis test, a nonparametric analog of analysis of variance, was used to assess for a statistically significant difference in at least one utility class compared with remaining terminologies. The Wilcoxon Rank-Sum test, a nonparametric analog of the student *t* test, was used to assess for a significant difference between the classes of each pair of two candidate terminologies. Both tests used a level of significance (α) of .05 and were performed separately for the 100 random and the 100 most frequent LOINC codes.

### Methods: Hypothetical Firing Scenarios

We devised seven hypothetical clinical cases that specified current and previous exams for which *proximate* or *similar* alerts should be fired based on anatomic location. These hypothetical scenarios were purposely selected to illustrate the differences between the anatomic terminologies in supporting our use case. We tested each anatomic terminology to see if, as presently constructed, *proximate* or *similar* alerts would appropriately fire. We defined a *proximate* region as the body region(s) adjacent to the body region hypothetically being ordered, as specified in SANOPS. For example, the “neck” region has *proximate* regions of “head” and “chest.” If the current and prior exams in our hypothetical scenarios are in *proximate* regions, then a *proximate* alert should be fired. A *similar* alert would be fired if the current and previous exams had different LOINC codes but mapped to the same anatomic region.

## Results

### Anatomic Terminology Utility Scale Scoring: 100 Random LOINC CT Codes

Descriptive statistics for the anatomic terminology utility scale classes for the five terminologies mapping to anatomic regions for 100 random LOINC codes are given in Table 2. Mean anatomic terminology utility class ranks ranged from 1.82 for RadLex to 2.44 for LOINC, suggesting that moderate modifications and/or additional computing steps are required over SANOPS for these standard terminologies to support prior similar or proximate alerts. Kruskal-Wallis analysis showed that there was no statistically significant difference in rank of the five candidate terminologies (*P*=.30).

**Table 2:** Descriptive statistics of the anatomic terminology utility classes for each anatomic terminology’s mapping to 100 random Logical Observation Identifiers Names and Codes (LOINC) codes. There was no statistical significance in mean terminology utility class rank.

Descriptive statistic	LOINC^a^	LOINC/RSNA^b^	RadLex	FMA^c^	SNOMED CT^d^
Median terminology utility class	1	1	1	1	1
Mode terminology utility class	1	1	1	1	1
Mean terminology utility class	2.44	2.18	1.82	2.22	2.03
Standard deviation of mean	1.83	1.75	1.37	1.56	1.32
Kruskal-Wallis mean rank class	267.53	245.75	229.66	258.16	251.40

^a^LOINC: Logical Observation Identifiers Names and Codes.

^b^RSNA: Radiological Society of North America.

^c^FMA: Foundational Model of Anatomy.

^d^SNOMED CT: Systematized Nomenclature of Medicine Clinical Terms.

**Table 3:** Descriptive statistics of the anatomic terminology utility classes for each anatomic terminology’s mapping to 100 most frequent Logical Observation Identifiers Names and Codes (LOINC) codes.

Descriptive statistic	LOINC^a^	LOINC/RSNA^b,c^	RadLex^c^	FMA^c,d^	SNOMED CT^c,e^
Median terminology utility class	2	1	1	2	2
Mode terminology utility class	2	1	1	2	2
Mean terminology utility class	2.6	1.5	1.38	2.29	2.19
Standard deviation of mean	1.85	1.22	1.13	1.44	1.31
Kruskal-Wallis mean rank class	293.16	203.66	188.18	285.65	281.88

^a^LOINC: Logical Observation Identifiers Names and Codes.

^b^RSNA: Radiological Society of North America.

^c^LOINC/RSNA Playbook and RadLex terminologies both had significantly lower class ranks compared with other terminologies by Wilcoxon Rank-Sum analysis.

^d^FMA: Foundational Model of Anatomy.

^e^SNOMED CT: Systematized Nomenclature of Medicine Clinical Terms.

### Anatomic Terminology Utility Scale Scoring: 100 Most Frequent LOINC CT Codes

Descriptive statistics for the anatomic terminology utility scale classes for the five terminologies mappings to anatomic regions for the 100 most frequent LOINC codes are given in Table 3. Kruskal-Wallis analysis shows a significant difference in at least one terminology mean class rank from another in the group. Wilcoxon Rank-Sum analysis shows that both RadLex and LOINC/RSNA terminologies ranked significantly lower (and therefore closer to our ideal SANOPS terminology) compared with the other terminologies (*P*<.001 for both), but there was no statistical difference in the ranking of the RadLex and LOINC/RSNA terminologies compared with each other (*P*=.15). Low mean terminology utility class ranks for RadLex and the LOINC/RSNA Playbook suggest that few, if any, modifications or additional computing steps above those used for SANOPS would be necessary to use these terminologies to support our use case in most instances where an alert would involve a frequently performed exam. Higher utility ranks for the LOINC, SNOMED, and FMA suggest that more computer resources and/or terminology modification would be necessary to employ these terminologies for our cases.

### Hypothetical Alert Firing Scenarios

Table 4 shows seven illustrative scenarios where either *similar* or *proximate* alerts should be issued based on the anatomic location of current and previous exams. For each exam pair, the table indicates which anatomic terminologies would fire alerts based on the mappings and anatomic regions in each terminology.

In the first three scenarios, appropriate alerts would be fired using all terminologies (*similar* in the first example and *proximate* in the second and third). All terminologies have concepts for the regions specified by the exam codes, and these regions correspond with major anatomic SANOPS regions.

In the fourth scenario (prior “Head CT” and current “Paranasal sinus CT”), only LOINC/RSNA Playbook and RadLex would issue appropriate *similar* alerts. In the LOINC/RSNA Playbook and RadLex terminologies, “head” is specified as the *region imaged* and *body region* attribute, respectively. In LOINC, “paranasal sinuses” is nested under “skeletal system,” bypassing “head.” In FMA, “paranasal sinuses” is nested under “anatomic spaces,” bypassing “head.” In SNOMED CT, “paranasal sinuses” are nested under “head” in some hierarchies but bypass “head” in others; the “head” or “paranasal sinus” parent or child relation can be expressed in a “look-up” table, but this would require an additional computing step over using SANOPS alone.

In the fifth scenario (prior “Liver CT” and current “Kidney CT”), *similar* alerts are issued only with RadLex. “Abdomen” is the specified *body region* attribute of the both liver and kidney CT exam codes in RadLex. In the FMA, LOINC, and LOINC/RSNA Playbook anatomic terminologies, there are links from “liver” to “abdomen” but no links from “kidney” to “abdomen.” SNOMED CT has links from both “liver” and “kidney” to abdomen but also divergent links bypassing “abdomen.”

In the sixth scenario (prior “Cervical spine CT” and current “Neck CT”), no alert would be fired using any standard terminology in an unaltered state.

**Table 4:** Hypothetical alert firing examples. Exam pairs where either *similar* or *proximate* alerts should be issued based on anatomic locations. Check marks (**✓**) note where appropriate alerts would fire for each exam pair using the anatomic terminology specified in column heading in its unmodified state.

Alert scenario	Prior exam	Current exam	LOINC^a^	LOINC/RSNA^b^	RadLex	FMA^c^	SNOMED CT^d^
1	Head CT^e^ with intravenous (IV) contrast	Head CT without IV contrast	✓	✓	✓	✓	✓
2	Head CT with IV contrast	Neck CT without IV contrast	✓	✓	✓	✓	✓
3	Elbow-bilateral CT without contrast	Shoulder-right CT with contrast IV	✓	✓	✓	✓	✓
4	Head CT with IV contrast	Paranasal sinuses CT without IV contrast		✓	✓		
5	Liver CT	Kidney CT without and with contrast IV			✓		
6	Cervical spine CT	Neck CT without IV contrast					
7	Esophagus CT	Chest CT					

^a^LOINC: Logical Observation Identifiers Names and Codes.

^b^RSNA: Radiological Society of North America.

^c^FMA: Foundational Model of Anatomy.

^d^SNOMED CT: Systematized Nomenclature of Medicine Clinical Terms.

^e^CT: computed tomography.

In LOINC, LOINC/RSNA Playbook, and RadLex, the major *body region* is “cervical spine” rather than “neck,” and there are no links from “cervical spine” to “neck.” FMA and SNOMED CT both contain links from “cervical spine” to the “neck” region but also links bypassing neck.

In the seventh scenario (prior “Esophagus CT” and current “Chest CT”), no alert would be fired using any standard terminology in an unaltered state. “Esophagus” is not nested under any *body region* in LOINC, LOINC/RSNA Playbook, or RadLex terminologies. In SNOMED CT, there are links from “esophagus” to “chest,” but there are also edge links bypassing “chest.” In FMA, there are circuitous edge links from “esophagus” to “chest” involving a mix of *has_part* and *part_of* links, necessitating a custom algorithm to appropriately fire a *similar* alert.

## Discussion

### Principal Findings

Our analysis of anatomic terminology utility classes for the 100 most frequent exam codes shows that RadLex and the LOINC/RSNA Radiology Playbook terminologies outperformed the other terminologies for our use case; however, our analysis for the 100 random LOINC codes showed no statistically significant difference in the performance of candidate standard terminologies with a range of utility class ranks of 1.82 to 2.44. Our analysis suggests the LOINC/RSNA Radiology Playbook and RadLex’s anatomic terminologies are suitable to support *proximate* or *similar* alerts for the most frequently performed CTs. The standard anatomic terminologies, as constructed at the time of this analysis, may have difficulties supporting our use case for uncommon CTs. Using the standard anatomic terminologies for issuing similar or proximate alerts would likely require the use of accessory look-up tables, modification of hierarchical relations, and/or application of SANOPS concepts and rules.

Our hypothetical test alerting scenarios illustrated how differences in the terminologies’ modeling affect each terminology’s fitness to support the alerting use case. In particular, the scenarios where no standard terminology supported appropriate alerts were selected to illustrate the difficulties of using existing terminologies, as is, for this use case.

The primary purpose of this study was to determine whether any of the standard anatomic terminologies in an unaltered state could approximate the performance of SANOPS and could potentially support a prior CT alerting system. We also wanted to assess and compare each of the standard terminologies to ascertain the effort required to adapt them for our use case. From our analysis, given the close approximation of utility classes for the LOINC/RSNA Playbook and RadLex anatomic frameworks to SANOPS for the most frequently performed CT exams, considerably less effort would be required to adapt these terminologies in their unmodified state for our use as compared with FMA or SNOMED CT.

Presently, we are collaborating with developers of the LOINC/RSNA terminology standard. In part influenced by our feedback, they are revising the modeling of the *region imaged* attribute such that all exams are assigned one of 11 discrete values (head, neck, chest, breast, abdomen, pelvis, extremity, upper extremity, lower extremity, whole body, and unspecified) [34]. This new modeling will enable us to leverage *region imaged* attribute to support our use case. As SANOPS’s partitioning of the extremities still differs from LOINC/RSNA, SANOPS extremity concepts will still be used to augment LOINC/RSNA until such a time that LOINC/RSNA can fully support our use case.

It should be noted that none of the five standard anatomic terminologies contained adjacency information between major body regions that we could utilize to support *proximate* alerts. Therefore, to use a standard terminology, we would have to use SANOPS model of proximity and adjacency to support an alerting system. FMA does have adjacency information expressed as *adjacent_to* and *bounded_by* relationship links. However, these relations are very granular (eg, esophagus *adjacent_to* thoracic aorta) and are not scalable to major body region adjacency relations. Additionally, adjacency relations are not expressed uniformly for all FMA concepts.

It should also be noted that many instances of FMA’s partonomy relations link organ systems to the body regions that contain them through homogeneous *part_of* links, but these relations are not expressed with the consistency needed to fully support our case (see esophagus to chest example above). The lack of comprehensive partonomy relations contributes to FMA’s overall higher utility class. SNOMED CT, by contrast, through its SEP relations had direct *is_a* links from organ systems to the body regions containing them in all observed instances. However, SNOMED CT’s polyhierarchy and alternate divergent pathways bypassing the body regions containing the organ systems may result in errors in linking organs back to body regions and contributed to its overall higher utility class.

### Future Considerations

In the near future, we plan to build a pilot alerting system and to expand it to encompass other imaging modalities such as MRI. We plan to use similar alerting rules for other modalities, as we have for CTs, to notify users of prior *same*, *similar*, or *proximate* exams. We anticipate that SANOPS concepts and rules can also be used to guide the utilization of any standard anatomic terminology to support *proximate* or *similar* alerts for other modalities.

### Limitations

The need for ongoing terminology maintenance would be a drawback to the long-term use of SANOPS as a stand-alone terminology. The standard terminologies are actively managed to support other use cases, and have active user communities that can enable more generalizable knowledge and sharing of resources. Using SANOPS in an operational system would require an ongoing effort to link any new standardized exam descriptions from LOINC used in our HIE to a SANOPS region. However, SANOPS is relatively simple with only 17 anatomic regions. On its own, SANOPS requires little maintenance. Also, SANOPS was never intended to be a stand-alone terminology. In our system, SANOPS extremity concepts are currently being used to augment anatomic concepts that extend beyond the current LOINC/RSNA Playbook.

The structure we chose for partitioning of extremities in SANOPS is not congruent with the standard anatomic terminologies and currently prevents direct mapping of SANOPS extremity concepts to “flattened” versions of these terminologies. However, SANOPS alerting rules in the extremities can be applied to standard terminologies by either mapping SANOPS extremity concepts to modified large extremity joint concepts in the standard terminologies or by partitioning the extremities by concepts already included in the standard terminology and then devising rules similar to SANOPS for *similar* or *proximate* alerts. For example, if a terminology contains concepts for “hip,” “thigh,” “knee,” “leg,” and “foot or ankle,” one could use these concepts to partition the lower extremity into categories for grouping exams by anatomic location and then set up alerting rules accordingly.

### Conclusions

Our analysis of the fitness of five standard anatomic terminologies to support *proximate* or *similar* alerts in a prior CT alerting system suggests that modifications of these terminologies based on our novel SANOPS anatomic framework may be necessary to fully support the use case. With increased interoperability and exchange of information across health systems, we foresee the need for anatomic frameworks to support *similar* or *proximate* alerts based on anatomic location. Our work with SANOPS may serve as guidance on methodology for using any terminology to support a prior imaging exam alerting system. Our evaluation may also inform the future assessment and use of these anatomic terminologies in other clinical applications.

## Abbreviations

CT: computerized tomography
FMA: Foundational Model of Anatomy
HIE: health information exchange
LOINC: Logical Observations Identifiers Names and Codes
MRI: magnetic resonance imaging
RPID: RadLex Playbook identifier
RSNA: Radiological Society of North America
SANOPS: Simple ANatomic Ontology for Proximity or Similarity
SNOMED CT: Systematized Nomenclature of Medicine Clinical Terms
